# Nose to Brain Delivery of Phenytoin Sodium Loaded Nano Lipid Carriers: Formulation, Drug Release, Permeation and In Vivo Pharmacokinetic Studies

**DOI:** 10.3390/pharmaceutics13101640

**Published:** 2021-10-08

**Authors:** Sreeja C. Nair, Kollencheri Puthenveettil Vinayan, Sabitha Mangalathillam

**Affiliations:** 1 Amrita School of Pharmacy, Amrita Vishwa Vidyapeetham, Kochi 682041, India; sreejacnair@aims.amrita.edu; 2 Division of Paediatric Neurology, Amrita Institute of Medical Sciences, Kochi 682041, India; vinayankp@aims.amrita.edu

**Keywords:** acute epileptic seizure, phenytoin sodium, olfactory epithelium, nanolipid carrier, nose to brain delivery

## Abstract

An acute epileptic seizure is a seizure emergency fatal condition that requires immediate medical attention. IV phenytoin sodium remains the second line therapeutic agent for the immediate treatment of status epilepticus. Phenytoin sodium formulated as nanolipid carriers (NLCs) seems to be promising as an intranasal delivery system for controlling acute seizures. Three different nanosized phenytoin sodium loaded NLCs (<50 nm, 50–100 nm and >100 nm) were prepared by melt emulsification and was further characterised. In vitro drug release studies showed immediate drug release from phenytoin sodium loaded NLCs of <50 nm size, which is highly essential for acute seizure control. The ex vivo permeation study indicated greater permeation from <50 nm sized NLC through the olfactory epithelium compared to thecontrol drug solution. Invivo pharmacokinetic studies revealed higher drug concentration in CSF/brain within 5 min upon intranasal administration of <50 nm sized phenytoin sodium NLCs than the control drug solution and marketed IV phenytoin sodium, indicating direct and rapid nose to brain drug transport through the olfactory epithelium. The study has shown that formulation strategies can enhance olfactory uptake, and phenytoin sodium NLCs of desired particle sizes (<50 nm) offer promising potential for nose to brain direct delivery of phenytoin sodium in treating acute epileptic seizures.

## 1. Introduction

A seizure emergency condition includes prolonged seizures or frequently visible seizures that require immediate medical attention. Seizures extending for more than five minutes are treated analogously to extremely severe and fatal seizures called status epilepticus. IV benzodiazepines including lorazepam, midazolam and diazepam remain as first-line therapeutic agents for the immediate treatment of status epilepticus once the patient reaches the hospital, while second line-therapy includes IV phenytoin sodium or fosphenytoin. The availability of benzodiazepines suitable for administration by alternative routes (other than IV and IM) such as rectal, buccal and intranasal offers additional advantages, facilitating their use immediately and outside of the hospital. In fact, these benzodiazepines are shortacting, with an elimination half-life of 1.5 h; thus, a second dose is mostly needed after 5 min or second-line Anti Epileptic Drugs (AED) have to be started immediately. When using benzodiazepines, there is an increased risk of fatal respiratory failure or arrest, which increases with frequent repeated doses [1,2,3].

Phenytoin sodium is a well-established and cost-effective traditional drug possessing long biological half-life of 15–22 h with a longer period of action (24 h). It does not produce any CNS depression or sedation compared to other AEDs [4]. It is extensively bound to plasma proteins, namely albumin (90% protein bound), and to other components of tissues. It shows an apparent distribution volume (V_d_) of 0.6 to 0.9 L/kg. Hepatic microsomal enzymes primarily metabolize phenytoin. Isoforms of the CYP2C subfamily are involved in the metabolism of phenytoin. It is extensively metabolized into an inactive para hydroxylated form called (4-hydroxyphenyl) -5 phenyl-hydantoin (HPPH metabolite) in the liver mediated by the cytochrome P450 2C19 (CYP2C19) enzyme system. At therapeutic drug concentrations, these enzyme systems responsible for metabolizing phenytoin become saturated. Hence, a constant amount of drug is metabolized and subsequent small increases in dose may increase the half-life and result in substantial increases in plasma drug concentration. There are disproportionate increases in steady-state drug levels if the dosage is increased by 10% or more, ultimately resulting in phenytoin toxicity. The hydroxylated product further undergoes glucuronide conjugation. Due to the saturable enzyme system present in the liver, it is of limited capacity. Interindividual variability is observed in plasma phenytoin concentrations after enzyme saturation. Thus, therapeutic drug monitoring (TDM) is needed for this drug [5,6,7]. The other disadvantages of phenytoin sodium include fatal hypotension and arrhythmias, and it is available as tablet and injections only, limiting its use outside the hospital setting in acute conditions. Moreover, the high alkalinity of available phenytoin sodium IV injection (pH 12) causes severe venous irritation and can only be provided with slow intravenous push at a concentration of 50 mg/min. Even at such lower infusion rates, profound hypotension is observed with unstable blood pressure or shock. Due to the above issues, this method has slow onset of action of around 30 min, and a large dose of 1–1.5 g (15–25 mg/kg dose) is necessary in order to achieve clinical effects due to the insufficient and inadequate delivery of phenytoin sodium to the brain, which ultimately results in potential peripheral toxic side effects. Considering the aforementioned limitations, as well as its unpredictable non-linear pharmacokinetic profile, clinicians have started prescribing other AEDs that are costlier than phenytoin, and undesirable CNS depressant effects are induced. Hence, newer formulations are highly essential for this affordable and well-established AED in order to bring it back to the clinic for utilization in acute epileptic emergencies [8,9].

In fact, several studies have shown a direct transport route from the olfactory region of the nasal cavity to the central nervous system in animal models, with no prior absorption into systemic circulation where there is an absence of BBB or even thinner barrier in the brain-olfactory interphase [10,11,12]. In nasal drug delivery, the brain and nose compartments are connected to each other via the olfactory route and via peripheral circulation, and drugs reach the brain without any interruption. Upon instillation of drugs from the nasal cavity, it first comes in contact with the nasal mucosa. There exist two pathways: The trigeminal pathway and olfactory pathway in nasal mucosa comprises respiratory mucosa and olfactory mucosa. The former consists of plenty of vascular capillaries. Either the drug is cleared out by mucociliary clearance or the drug enters into systemic circulation and then crosses the blood–brain barrier to reach the brain. The latter follows a direct connection into the brain through the olfactory lobe, bypassing the BBB. The olfactory region of nasal mucosa that provides a direct connection between nose and brain can be exploited for targeting CNS acting drug molecules [13]. Formulating drugs into particulate carriers with smaller particle size, low molecular weight and lipophilicity enables targeted deposition and the retention of the drug onto the olfactory epithelium for subsequent nose to brain direct transportations. Nanostructured lipid carriers (NLC) are binary structures in which the lipid matrix consists of a mixture of solid lipid and liquid lipid. In comparison to other conventional nanosystems, lipid nanocarriers offer numerous advantages, including ease of large-scale production, biocompatible and biodegradable materials, low toxicity potential, and the possibility of modified drug release, drug solubility enhancement and the incorporation of both hydrophilic and lipophilic drugs. Due to their imperfect crystal structure, NLCs offer higher drug loading capacity and can prevent drug expulsion by preventing lipid crystallisation during manufacturing and storage [14]. Moreover, the addition of a large amount of liquid lipids enables the NLC system to achieve smaller particle size, which remains a good strategy to enable size-dependent direct transport of drugs through the olfactory nasal epithelium [15,16,17]. The synergistic effect of size tailored nanotechnological methods and direct delivery via nose-to-brain brings new hope to patients with epileptic syndromes. Our study aimed to enhance brain delivery of phenytoin sodium by direct nose to brain transport through the olfactory epithelium with a faster onset of action and reduced dose-related peripheral side effects for treating acute epileptic seizures [18,19,20].

## 2. Materials and Methods

### 2.1. Materials

Phenytoin sodium API was purchased from Sigma Aldrich, St. Louis, MO, USA, and was used as received. Oleic acid was purchased from Loba Chemie (Mumbai, India). Cholesterol A.R and Pluronic-F-188 (Poloxamer) were procured from Nice Chemicals (Kochi, India). Dialysis membrane (12,000–14,000 Dalton) was from Sigma Aldrich (Bangalore, India). Fibroblast (L929) cells were purchased from National Center for Cell Sciences, Pune, India, and Human Brain Capillary Endothelial Cells (HBCEC)[ATCC-CRL-3245] were received from LGC Promochem India Pvt. Ltd. (Bangalore, India). Ethanol, acetone, potassium di hydrogen ortho phosphate, ortho phosphoric acid, diethyl ether, high pressure liquid chromatography (HPLC) grade methanol, acetone and acetonitrile were purchased from Merck Chemical Company, Mumbai, India.

### 2.2. Methods

#### 2.2.1. Preparation of Phenytoin Sodium (PS) Loaded NLCs

**Figure 1:**
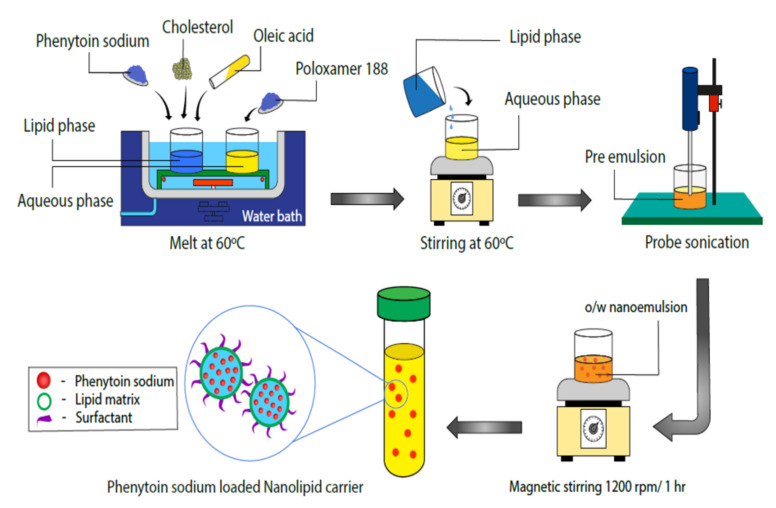
Schematic representation of method of preparation of phenytoin sodium loaded NLC.

Phenytoin sodium loaded NLCs were formulated by melt emulsification with the ultrasonication method using cholesterol (as solid lipid), oleic acid (as liquid lipid) and poloxamer188 as the polymer. Different sized NLCs were prepared by varying probe sonication time to study the influence of particle size on intranasal olfactory uptake. To the preheated mixture of lipids, i.e., cholesterol and oleic acid, phenytoin sodium was added and maintained at 60 °C in a water bath. The oil phase was slowly added to a preheated aqueous phase containing 1% *w*/*v* of poloxamer188 in deionized water at 60 °C and magnetically stirred at 2000 rpm for 20 min. The obtained pre-emulsion was then ultrasonicated by using a probe sonicator (Sonics/CV18/2014) to produce an o/w nanoemulsion. The probe sonication parameters were different (sonicating time was 15 min at 30% amplitude for a cycle of 8 s on and 2 s off for preparing >100 nm sized phenytoin sodium NLC; similarly, 20 min at 30% amplitude for a cycle of 8 s on and 2 s off for preparing 50–100 nm sized phenytoin sodium NLC, and it was 25 min at 40% amplitude for a cycle of 8 s on and 2 s off for formulating optimized <50 nm sized phenytoin sodium NLCs) for different sized phenytoin sodium NLCs. Finally, o/w nanoemulsion obtained was cooled down to room temperature while stirring at 1200 rpm for about 1 h in a magnetic stirrer. The obtained NLCs were filtrated through a 0.45 µm membrane filter to remove the unincorporated PS aggregates. The resulting NLCs were finally washed three times with purified water [21]. All NLC formulations were designed to contain 4 mg/mL of the drug. The schematic representation of the method of preparation of PS loaded NLC is shown in Figure 1.

#### 2.2.2. Characterization of Phenytoin Sodium (PS) Loaded NLCs

##### Determination of Particle Size, Polydispersity Index (PDI) and Surface Potential

The mean hydrodynamic nanoparticle size, distribution, PDI and surface potential of different sized NLCs were determined by the dynamic light scattering method in a particle size analyzer (DLS-ZP/Particle Sizer Nicomp TM 380 ZLS Malvern, Worcestershire, UK). Formulations were previously diluted 20-fold with double distilled water at room temperature. A sample volume of 500 μL was employed for size distribution and PDI analysis. A volume of 1000 μL was used for the determination of surface potential. All the measurements were performed in triplicate. The average value from the measurement of the diameter of three NLC formulations was then calculated [22,23,24].

##### Fourier Transform Infrared (FTIR) Spectroscopy Study

FTIR analyses of phenytoin loaded with NLC, pure drug and the excipients were conducted to study the possible interactions of drugs with the excipients using a FT Infrared Spectrophotometer (Shimadzu-8400 S, Kyoto, Japan) with the potassium bromide (KBr) pellet method.

##### Transmission Electron Microscopy

The surface morphological properties of the particles were analysed by the Transmission Electron Microscopy technique (TEM-Tecknai G2, FEI, 200 kV, Tokyo, Japan). An NLC drop was diluted 50 times with water and spread over a carbon film-coated 200 mesh copper grid that was held for about 3 min. A drop of 2% *w*/*w* phosphotungstic acid was placed on the grid for a maximum of 30 s, and a filter paper was used to remove excess droplets. The grid was then air-dried for approximately 2 h and then used for microscopic analysis.

##### Determination of Drug Entrapment Efficiency (EE) and Drug Loading Efficiency (LE)

The percentage entrapment efficiency of the drug in the NLC was taken into account by homogenizing the prepared different sized drug-loaded NLCs using 10 mL methanol for 2 h followed by centrifugation performed in a high-speed refrigerated centrifuge (HERMLE/232HK/2014) at 15,000 rpm for 20 min at 6 °C. The supernatant obtained after centrifugation was quantified by using the validated HPLC technique (LC 2010A HT SHIMADZU) at 220 nm. The total amount of drug entrapped into the NLC system can be directly quantified [25,26].

% Entrapment Efficiency = (Amount of drug entrapped/Amount of drug taken initially) × 100

For the determination of the LE, dry weight of the lyophilized form of NLC (total carrier system) was measured, and % loading efficiency was calculated as the following.

% Loading Efficiency = (Amount of drug entrapped/Total weight of NLC) × 100

#### 2.2.3. In Vitro Drug Release Study

The release profiles of phenytoin sodium (PS) from three different sized NLCs (<50 nm phenytoin sodium loaded NLCs, 50–100 nm phenytoin sodium loaded NLCs and >100 nm phenytoin sodium loaded NLCs) were studied by using the cellophane membrane barrier method. A dialysis cellulose membrane with a molecular weight cut off from 12,000–14,000 Dalton was tied to one end of the open-ended tube, and 1 mL of NLCs equivalent to 4 mg phenytoin sodium was transferred into it through the other end [27]. The open tube containing NLC was immersed in a 100 mL methanol-phosphate buffer (pH 7.3) receptor compartment in a 70:30 ratio. The temperature was kept constant at 37 ± 0.5 °C, and the receptor fluid was agitated in a magnetic stirrer at 100 rpm. Throughout the test, the sink condition was maintained. A sample (0.5 mL) was withdrawn at 1, 3, 5, 7, 9, 11, 13, 15, 30 and 45 min, respectively, from the receptor compartment and was replaced with the same amount of fresh solvent. The amount of released phenytoin sodium was determined by using the validated HPLC method. All the experiments were performed in triplicate. C18 column was used for HPLC analysis (LC 2010A HT SHIMADZU, Shimadzu, Kyoto, Japan) as the stationary phase, and a mixture of methanol-phosphate buffer (pH 7.3) in 70:30 ratio was used as the mobile phase. The injected volume was 10 μL, the wavelength was set at 220 nm and the user flow rate was 0.7 mL/min. The cumulative percentage drug release was then plotted against time. The release data were integrated into different drug release kinetic models, such as Zero order, First order and Higuchi and Korsmeyer Peppas models, in order to determine the release kinetics of phenytoin sodium. The best model was determined by using the values of the exponent (*n*) to determine the most fitting model to explain the release mechanism [28].

#### 2.2.4. Ex Vivo Permeation Study

The ex vivo permeation comparison study using Franz diffusion cells was carried out for 1 h for <50 nm phenytoin sodium loaded NLCs, 50–100 nm phenytoin sodium loaded NLCs, >100 nm sized phenytoin sodium loaded NLCs, control drug solution (drug in pH 6.6 buffer) and intranasal midazolam spray marketed formulation using freshly excised bovine nasal mucosa by separating the upper olfactory epithelium and lower trigeminal epithelium [29]. The olfactory and trigeminal mucosa surface area exposed to the formulation treatments was 2.54 cm^2^, and the volume of the receptor fluid was 7 mL. Following the hydration of the mucosa, the mucosal epithelium was placed between the diffusion cell donor and receptor compartments. The amount of 1 mL of NLCs or other formulations equivalent to 4 mg drug was applied to the respective dorsal surface of mucosa in the donor compartment, while the receptor compartment was filled with a 70:30 methanol-phosphate buffer (pH 6.6) mixture magnetically agitated at 100 rpm. The diffusion cell was thermostated at 37 ± 0.5 °C. A volume of 0.5 mL was withdrawn from each Franz diffusion cell’s receptor compartment at 1, 3, 5, 7, 9, 11, 13, 15, 30 and 60 min intervals and was immediately replaced by the same volume of fresh methanol-phosphate buffer mixture to enable sink conditions. All the experiments were performed in triplicate. The withdrawn samples were then sonicated followed by filtration by passing it through a 0.22 µm filter membrane. The cumulative amount of drug permeated through olfactory and trigeminal epithelium was quantified separately by the validated HPLC method (LC 2010A HT SHIMADZU) at 220 nm using a C18 column and a mixture (70:30 ratio) of methanol-phosphate buffer (pH 7.3) as the mobile phase. The injected volume was 10 μL, and the flow rate was fixed at 0.7 mL/min. The total amount of drug permeated/cm^2^ versus incubation time was drawn graphically, and the slope of the graph corresponds to the steady-state flux (*J*) value [30,31].

#### 2.2.5. In Vitro Cytocompatibility Studies by MTT Assay

In vitro cytocompatibility of <50 nm sized and >100 nm sized bare NLCs, <50 nm sized and >100 nm sized phenytoin sodium loaded NLCs bare drug in nasal pH buffers were carried out on L929 fibroblasts cell lines and human brain capillary endothelial cell lines (HBCECs) by an MTT [3-(4, 5-dimethylthiazole-2-yl)-2, 5 diphenyl tetrazolium] assay. The culture medium used to maintain cell lines was the modified Dulbecco Eagles Medium (DMEM) fortified with 10% fetal bovine serum (FBS). In the CO_2_ incubator, the cultivation flasks are maintained with 5% CO_2_. The cells were subcultured by the use of Trypsin-EDTA to detach from the flask. Different sample concentrations (0.2 mg/mL and 0.4 mg/mL respectively) were prepared for the experiment by the dilution of stock solution/formulation (1 mg/mL) using the prepared medium. The cells were counted and seeded at a density of 10,000 cells/cm^2^ in 96-well plates. After obtaining suitable confluency, the cells were treated with the appropriate samples for a specified period of time (24 h). Cells treated with Triton served as the negative control, and media with untreated cells acted as the positive control. The cells were treated with MTT and again kept in a CO_2_ incubator for 4 h for the formation of formazan crystal. Finally, the crystals formed were solubilized by using a solubilization buffer, and the optical density was measured at 570 nm using an Elisa plate reader (Bio Tek Power Wave XS, BioTek Instruments, Winooski, VT, USA) [32,33]. Triplicate samples were analysed for each experiment.

#### 2.2.6. Human Brain Capillary Endothelial Cell (HBCEC) Uptake Study Using Fluorescent Microscopy

The cell uptake study was carried out using the fluorescent microscopy technique. The <50 nm sized bare NLCs below 50 nm sized phenytoin sodium loaded NLCs were tagged with Rhodamine 123 to make NLC fluorescent. Rhodamine 123 solution was initially prepared in ethanol (1 mg/mL). From each NLC formulation, 5 mL was taken and stirred with 100 μL of Rhodamine 123 solution in a magnetic stirrer for 3 to 4 h to facilitate tagging of Rhodamine with NLC. HBCEC cell lines were seeded into acid-etched cover slips mounted in 12 well plates (30,000 cells per cover slip). After 24 h of incubation maintained at 37 °C in a 5% CO_2_ incubator, when the cells were observed to be well attached to the cover slip, the medium was removed. The wells were then washed with PBS buffer three times. This was followed by the addition of the required volume of rhodamine tagged NLC samples (concentration of 1mg/mL) into the well and was incubated for 2 h. The cover slips were again washed with phosphate buffer, and the fixation of cells was performed with 4% paraformaldehyde (PFA). An amount of 100 µL of DAPI (4′,6-diamidine-2-phenylindole)stain (1:15 ratios in PBS) and 5 µL of Actin stain were added after. The air-dried cover slips were then mounted on a glass slide using DPX as a mounting agent, and they were observed appropriately by using various filters under a fluorescent microscope (Olympus–BX 51, Olympus Corporation, Tokyo, Japan).The obtained images were merged properly [34,35].

#### 2.2.7. In Vivo Pharmacokinetic Study of Phenytoin Sodium NLCs in Wistar Rats

Female Wistar rats of 8 to 12 weeks age (200–250 g body weight) were used for in vivo pharmacokinetic study. All animal experiments were performed after the receiving approval from the Institutional Animal Ethics Committee (IAEC), Amrita Institute of Medical Sciences, Kochi, Kerala, India (IAEC Ref. No. IAEC/2018/2/12), and all the guidelines of animal handling and experimentation were strictly follows. The study comprised of 7 groups consisting of 200 rats; each treatment group had 30 rats for a total 5 set time intervals for a total 1 h. Before drug administration, the animal was anaesthetized using 5% isoflurane in an anaesthesia chamber for not less than 1 min. For nose to brain intranasal administration in rats, volumes equivalent to 100 μL each of saline solution, <50 nm sized phenytoin sodium NLCs, >100 nm sized phenytoin sodium NLCs, control drug solution and 160 μL of intranasal midazolam marketed formulation, each containing 800 μg drug (Table 1) were instilled into the nares towards the roof of the nasal cavity targeting the olfactory mucosa region. The rats were held from the back, in a slanted position, during intranasal administration and thereafter for two minutes. For comparison, the sixth group was injected with 16 μL of intravenous (i.v.) phenytoin marketed formulation containing 800 μg of drug through the tail vein. At 5, 10, 15, 30 and 60 min, the respective rats were euthanized by keeping them in a carbon dioxide inhalation chamber with a flow rate of 3 L/min for 5 min. The CSF samples were collected by the cisterna magna puncture method and blood samples (4 mL) by the cardiac puncture method, and plasma was then separated. For the collection of CSF, the neck of the rat’s skin was shaved, and the animal was then placed on the stereotaxic instrument. The head was then secured with the help of head adaptors. The surgical site was swabbed with 10% povidone iodine, followed by 70% ethanol (repeated 3 times). An incision was made in the skin over the occipital bone, and the first layer of muscle was cut. After exposing the atlanto-occipital membrane, 80–100 µL CSF was taken through the membrane by inserting a 30-gauge needle attached to an insulin syringe. The CSF is transferred into a pre-marked 1 mL Eppendorf tube, and the sample was centrifuged at 10,000 rpm for 20 min, and 50 µL supernatant was collected for drug quantification [36]. After processing, the drug concentrations in plasma and CSF were estimated for pharmacokinetic evaluation.

Subsequently, the rats were subjected to dissection of the entire brain and other peripheral organs in order to study drug distribution in major tissues. The major vital organs such as liver, kidney, lungs, spleen, pancreas and heart, etc., were isolated, washed twice using normal saline and freed from adhering tissue or fluid. After the extraction procedure, all biological samples were analysed for drug content by the validated HPLC method at 220 nm. A mixture of diethyl ether and a mobile phase (methanol:pH buffer) was used as a solvent for extraction of phenytoin sodium from CSF, plasma and entire brain and other peripheral organs homogenates. Aliquots of 10 μL of each processed sample after filtration through 0.22 µm syringe filter were injected into the chromatographic system for HPLC (LC 2010 A HT SHIMADZU Corporation, Kyoto, Japan) analysis using aC18 column, an isocratic elution having mobile phase (methanol:pH 2.8 buffer in 60:40 ratio) pumped at a flow rate of 0.7 mL/min and the temperature of the column was set at 35 °C. The phenytoin sodium concentration was detected at a wavelength of 220 nm with a total running time of 10 min. The peak area under the absorption time curve was noted for each sample. Using Phoenix Win Nonlin ^®^ software (Phoenix Software version 8, Certara, Princeton, NJ, USA), various pharmacokinetic parameters of phenytoin sodium such as Cmax, Tmax, AUC, half-life, mean residence time and elimination rate constant were determined by applying non-compartmental pharmacokinetic analysis [37].

#### 2.2.8. In Vivo Nasal Toxicity Study

Histopathological analysis of isolated rat olfactory mucosa and olfactory bulb treated with <50 nm phenytoin sodium loaded NLC was also performed to assess any potential local toxic effects in the nasal olfactory region and was compared with that of the control drug solution (drug in PBS pH 6.8) treated group. All the animal experiments were performed after the approval from Institutional Animal Ethics Committee (IAEC), Amrita Institute of Medical Sciences, Kochi, Kerala, India (IAEC Ref. No. IAEC/2018/2/12). Four female Wistar rats (total two groups) of 180–220 g of body weight and 7–9 weeks of age were randomly selected for the nasal toxicity study. Before drug administration, the animal was anaesthetized using 5% isoflurane in an anaesthesia chamber for not less than 1 min. For nose to brain intranasal administration in rats, a volume equivalent to 100 μL of <50 nm sized phenytoin sodium NLC as well as control drug solution was instilled into the nares towards the roof of the nasal cavity targeting the olfactory mucosal region. The rats were held from the back, in slanted position, during intranasal administration and thereafter for two minutes. After 1 h of intranasal administration of various formulations, the animals were sacrificed by keeping them in a carbon dioxide inhalation chamber having a flow rate of 3 L/min for not less than 5 min. The olfactory mucosa and the olfactory bulb were then carefully removed from each nostril with the help of surgical tools and dissection microscope, which was then immediately washed and kept in ice cold Ringer’s solution. The isolated regions were washed with distilled water and preserved in 10% formalin in saline solution. Sections of 50 µm were taken using a cryotome (Leica CM 1505 S, Leica Biosystems, Mumbai, India), and they were then stained with haematoxylin and eosin and then examined by using light microscope to reveal local toxicity in the nasal olfactory mucosa as well as olfactory bulb region [38].

#### 2.2.9. Statistics

All data were statistically analyzed using the one-way ANOVA test. A value of *p* < 0.05 was considered to be significant.

## 3. Results and Discussion

### 3.1. Preparation and Characterization of Phenytoin Sodium (PS) Loaded NLCs

The optimized stable NLCs prepared by the melt emulsification method composed of 80% oleic acid, 20% cholesterol as lipid phase and 1.5% *w/v* of poloxamer188 as the surfactant. By optimizing the probe sonication time at common amplitude, necessary sizing in the nano regime was achieved. It was found that the particle size of NLCs decreases with an increase in the duration of probe sonication, i.e., to achieve the desired size, sonicating time was 15 min for obtaining NLC of >100 nm size (124.56 ± 3.11 nm) and it was 20 min for preparing 50–100 nm sized phenytoin sodium NLC (80.0 ± 2.45 nm) and was 25 min for the <50 nm sized (32.59 ± 3.42 nm) phenytoin sodium NLCs. The prepared phenytoin sodium loaded NLCs were characterized for various physicochemical parameters. The average particle size of NLC was 32.59 ± 3.42 nm (PDI-0.289), 80.0 ± 2.45 nm (PDI-0.256) and 124.56 ± 3.11 nm (PDI-0.303) for the three different sized phenytoin sodium loaded NLCs. The PDI values obtained are found below 0.35, which indicates the uniformity of particle size. All the NLCs showed a negatively charged zeta potential (−16.5 ± 0.12 to −28.0 mV ± 1.87) due to the influence of negatively charged phospholipids which impart negative charge to the particle. The total amount of lipid and surfactant added in the formulation also influences the particle size. As the liquid lipid concentration increases, a decrease in particle size has been observed [39]. As per scientific reports, the smaller particle size <50 nm can easily travel across the olfactory nasal epithelium and can reach the brain within minutes [40]. Hence, we are assuming that the 32.59 ± 3.42 nm particle size obtained would be favorable for direct intranasal olfactory uptake.

**Figure 2:**
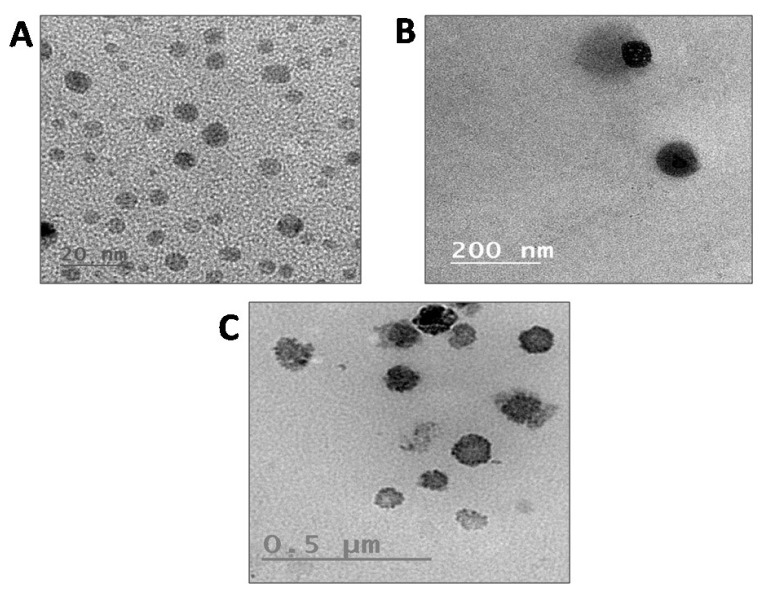
TEM images of <50 nm sized phenytoin sodium loaded NLC (**A**), 50–100 nm sized phenytoin sodium loaded NLC (**B**) and >100 nm sized phenytoin sodium loaded NLC (**C**).

**Figure 3:**
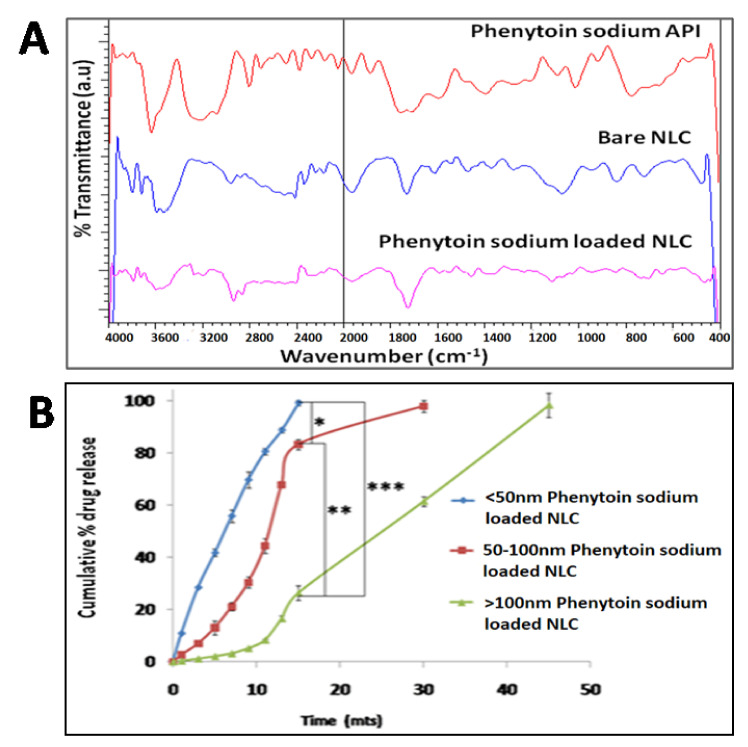
Characterization of NLC by FTIR analysis (**A**). In vitro drug release study of phenytoin sodium NLCs (**B**). The level of statistical significance is expressed as a *p*-value; * indicates *p* < 0.05, ** indicates *p* < 0.01, *** indicates *p* < 0.001.

TEM images (Figure 2A–C) revealed that the particles possessing spherical morphology and size were correlated with DLS results. FTIR spectra revealed sharp stretching vibrations for the NH group at 3300 and 3200 cm^−1^, aromatic C-H group at 3050 cm^−1^ and carbonyl group of phenytoin sodium was observed as stretching vibrations at 1700 and 1740 cm^−1^. The IR spectrum of poloxamer 188 was characterized by principal absorption peaks at 2881 cm^−1^ (C–H stretch aliphatic), 1348 cm^−1^ and 1107 cm^−1^ (C–O stretch). The diagnostic bands identified for cholesterol were the strong bands around 2929, 1463 and 1054 cm^−1^. For oleic acid, the peak in the band 1650–1742 cm^−1^ is the characteristic stretching vibration of the C=O group present in COOH, and the peak at 2911 cm^−1^ is due to asymmetric CH_2_ stretching. In phenytoin sodium loaded NLC, bands of phenytoin sodium, cholesterol and oleic acid were observed, indicating the presence of phenytoin in the NLCs (Figure 3A).

### 3.2. Determination of Percentage Entrapment Efficiency (EE) and Percentage Drug Loading (DL) 

The average percentage entrapment efficiency and drug loading were found to be 91.17 ± 4.48% and 39.43 ± 2.80%, respectively, for <50 nm sized phenytoin sodium loaded NLC, 87.70 ± 1.19% and 36.92 ± 4.71%, respectively, for 50–100 nm sized NLC, 81.35 ± 3.17% and 32.54 ± 1.27%, respectively, for >100 nm sized phenytoin sodium loaded NLCs. The finding showed that NLCs having lower particle size (<50 nm) have the highest entrapment efficiency and drug loading compared to larger size (>100 nm) phenytoin sodium loaded NLC. In an NLC based system, the lipophilicity of the drug and the addition of lipidic excipients used to prepare NLC make the formulation highly lipophilic, resulting in high EE enforcing its maximum entrapment inside the matrix. In addition, drug loading is dependent on particle size as this smaller particle sized NLCs can be easily and uniformly well dispersed inside the lipid matrix without aggregation resulting in high DL [41].

### 3.3. In Vitro Drug Release Study

The in vitro drug release study was performed using the dialysis membrane technique and showed an immediate drug release of 99.19 ± 1.07% for <50 nm sized NLCs, whereas 83.27 ± 2.01% drug release for NLCs having 50–100 nm size and 26.38 ± 2.93% release for >100 nm sized NLCs at the end of 15 min were observed (Figure 3B). A complete drug release was observed within 15 min for <50 nm sized NLCs, whereas phenytoin sodium NLCs having 50–100 nm size showed maximum drug release (97.95 ± 2.25%) at 30 min. In the case of >100 nm larger sized NLC, 98.36 ± 4.68% drug release after 45 min was observed. This immediate release is highly essential for acute seizure control in epilepsy. These smaller sized nanosystems favored a shorter average diffusion path for the drug molecules that are entrapped in the matrix, allowing faster diffusion and resulting in higher drug release from <50 nm NLC compared to >100 nm NLC. Moreover, the smaller sized nanosystem contributes to faster polymer degradation or erosion, which results in increased drug diffusion from the polymer matrix. The obtained in vitro release data of phenytoin sodium loaded NLCs were fitted to different kinetic models. The coefficient of regression (R^2^ value) of different kinetic models indicates that the drug release follows zero-order kinetics, which is better fitted with the Korsmeyer peppas model with n value more than one, indicating that the drug release mechanism follows non-Fickian transport [42].

### 3.4. Ex Vivo Permeation Study

**Figure 4:**
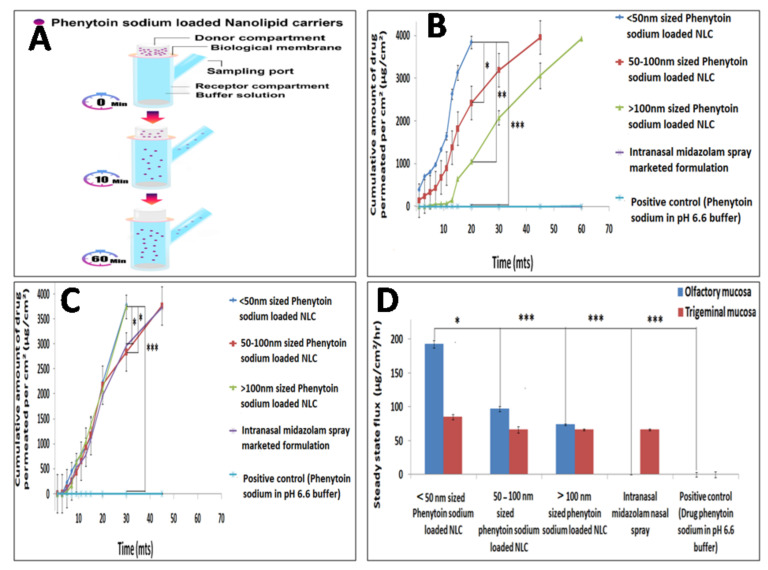
Theschematic representation of ex vivo permeation study of phenytoin sodium NLCs (**A**), ex vivo permeation study of phenytoin sodium NLCs using olfactory mucosa (**B**) and trigeminal mucosa (**C**). Steady-state flux determination of various intranasal formulations (**D**). The level of statistical significance is expressed as a *p*-value; * indicates *p* < 0.05, ** indicates *p* < 0.01, *** indicates *p* < 0.001.

The cumulative olfactory permeation through <50 nm sized phenytoin sodium NLC was found to be 3843.16 µg/cm^2^ at the end of 20 min, which showed a size dependent faster permeation compared to other formulations: from 50 to 100 nm sized NLC, it was found to be 3962.56 µg/cm^2^ in 45 min; from >100 nm sized NLC, it was 3929.34 µg/cm^2^ in 60 min; from the control drug solution, it was 1.09 µg/cm^2^ in 60 min; and no drug permeation from intranasal midazolam spray marketed formulation was observed at the end of 60 min. Similarly, the cumulative trigeminal mucosal permeation from <50 nm sized NLC was found to be 3775.12 µg/cm^2^ at the end of 45 min, which also showed a faster permeation compared to other formulations: from 50 to 100 nm sized NLC, it was found to be 3769.66 µg/cm^2^; from >100 nm sized NLC, it was 3752.76 µg/cm^2^; from intranasal midazolam formulation, it was 3732.04 µg/cm^2^; and from the control drug solution, it was 5.68 µg/cm^2^ (Figure 4A–C). The <50 nm phenytoin sodium NLC also showed higher steady-state flux compared to the control drug solution (Figure 4D). Since the study is focused on treating acute seizure conditions, the higher drug permeation occurring for <50 nm sized phenytoin sodium NLC through the olfactory epithelium will be beneficial due to its small size as well as lipidic nature of NLC and also receive protection from metabolic enzymes localized in the nasal mucosal cavity, whereby it reaches the brain rapidly and releases drug within minutes in order to obtain a rapid onset of action, which is not feasible through the trigeminal mucosal route due to its high vascularity. Furthermore, the permeation enhancing effect of surfactant poloxamer, which have a direct effect on the cell membrane, favors faster permeation of the drug by creating pores in the olfactory mucosa. This further result in lipid bilayer disruption by providing a better platform for effective permeation of drug across the olfactory epithelium for direct nose-to-brain delivery [43].

### 3.5. In Vitro Cytocompatibility Studies

**Figure 5:**
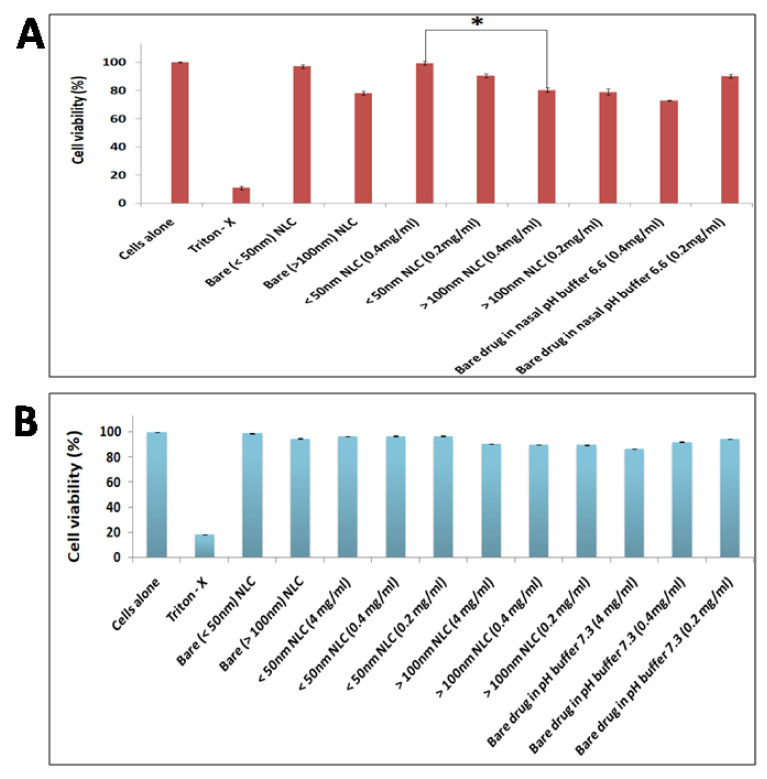
Cytocompatibility studies of the prepared Phenytoin sodium loaded nano lipid carriers and bare drug in (**A**) L929 and (**B**) HBCEC cell line. The level of statistical significance is expressed as a *p*-value; * indicates *p* < 0.05.

In vitro cytocompatibility studies of different formulations were performed on L929 fibroblast cells as well as HBCEC cell lines by MTT assay. The obtained results confirmed the non-toxic nature of NLC. All the NLCs showed cell viability of 75–99% for L929 cells and 85–99% for HBCEC cell lines (Figure 5A,B), respectively, after 24 h incubation. The results indicated that prepared NLCs are biocompatible.

### 3.6. Human Brain Capillary Endothelial Cell (HBCEC) Uptake Using Fluorescent Microscopy

**Figure 6:**
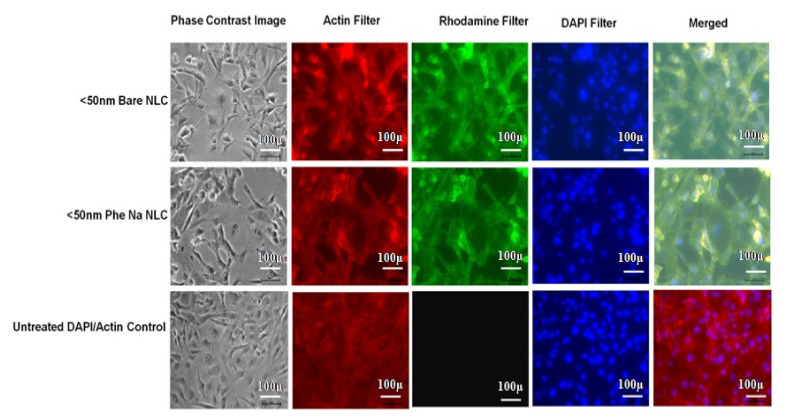
Fluorescent microscopic images showing the uptake of NLC by HBCEC cell line.

The cell uptake of phenytoin sodium NLCs by human brain capillary endothelial cells (HBCEC) was determined by using fluorescent microscopy. To detect the NLC particle using fluorescent microscopy, we labelled the control as well as the <50 nm sized bare NLCs and the <50 nm sized phenytoin sodium NLCs with a lipophilic fluorescent Rhodamine 123 dye. After removing the unconjugated rhodamine fraction by centrifugation process, the rhodamine bound NLCs were taken for BCEC cell uptake studies. After staining the cells by using Actin and DAPI followed by fluorescent microscopy imaging, internalizations of rhodamine tagged <50 nm sized bare NLCs and drug loaded NLCs by HBCEC cells were determined. Actin provides red colour stain to the cytoskeleton of the cell, and DAPI stains the nucleus of the cells as blue. The combined control cell images showed the cytoskeleton of cells having rounded nucleus. In rhodamine -tagged NLC treated cell lines, the presence of rhodamine-conjugated particles is observed in green colour, and the combined images show the presence of particles in the entire BCEC cells (Figure 6). The intensity of green color was prominent for <50 nm sized NLC treated cells, which confirms that the size of the particle plays an important role in cell uptake. This phenomenon may result from the higher affinity of NLC’s biocompatible lipid materials for the HBCEC cell membrane and the nano size of particles [44].

### 3.7. In Vivo Pharmacokinetic Study of Phenytoin Sodium NLCs in Wistar Rats

**Figure 7:**
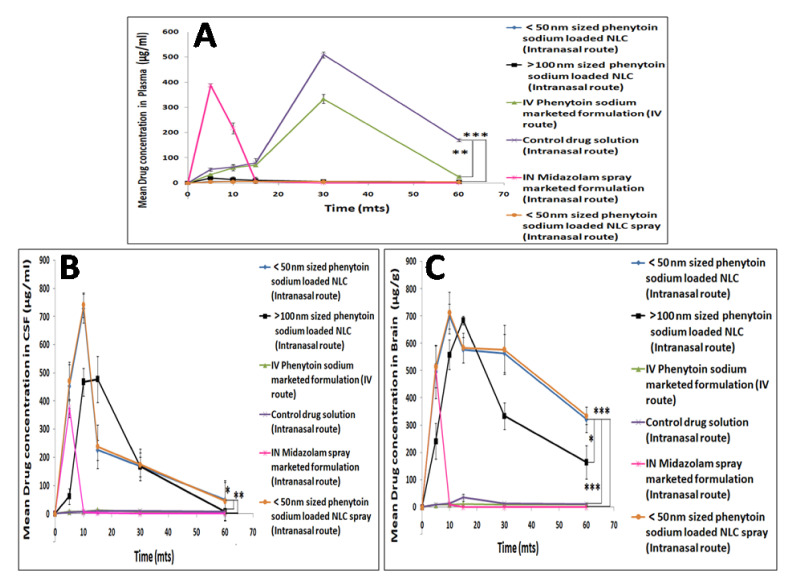
Mean plasma (**A**), CSF (**B**) and brain (**C**) drug concentration profile following intranasal administration of phenytoin sodium NLCs, control drug solution, midazolam marketed formulation and IV administration of phenytoin sodium marketed formulation. The level of statistical significance is expressed as a *p*-value; * indicates *p* < 0.05, ** indicates *p* < 0.01, *** indicates *p* < 0.001.

In the in vivo pharmacokinetic study, the drug concentration was measured in plasma, CSF and the brain at regular intervals up to 1 h by using the validated HPLC method, and area under the curve (AUC) was also calculated. The HPLC method was fully validated for linearity. The linear response range for the plasma and CSF sample was found to be 20–90 μg/mL and 100–600 μg/mL, respectively, whereas the linear response range obtained was 50–700 μg/g for the entire organ tissues sample. Figure 7A,B shows the plasma and CSF concentration-time profiles after intranasal administration of <50 nm phenytoin sodium NLCs, >100 nm phenytoin sodium NLCs, control drug solution, midazolam spray marketed formulation and IV administration of phenytoin sodium marketed formulation. The in vivo pharmacokinetic study showed that higher drug concentrations were observed in CSF within 5 min of intranasal administration of <50 nm sized phenytoin sodium NLCs than compared to the intranasal control drug solution and marketed phenytoin sodium IV formulation. In addition, lower drug concentration was observed in plasma after 5 min of intranasal administration of optimized <50 nm NLC formulation compared to the control drug solution and marketed phenytoin sodium IV formulation. This indicates that there is minimal systemic absorption of drug-loaded NLCs via the intranasal route, confirming that uptake is not through the systemic pathway but through the olfactory epithelial perineural pathway [45]. In vivo brain retention study is of great importance since it is the target organ for drug action. The study revealed that <50 nm phenytoin sodium NLC showed higher drug retention in the brain compared to other organs within 5 min of intranasal administration (Figure 7C). A similar trend of enhanced brain retention is obtained for intranasal midazolam spray marketed formulation. However, the control drug solution and IV phenytoin sodium marketed formulation showed less drug retention in the brain compared to intranasal phenytoin sodium NLCs. The 30–40-fold increase in brain AUC_0–∞_ of phenytoin sodium from <50 nm sized phenytoin sodium NLC via the intranasal route in comparison to that of intranasal control drug solution and IV phenytoin sodium further confirms direct nose to brain transport via the olfactory region. Moreover, there is a notable difference in the brain AUC_0–∞_ value between <50 nm sized phenytoin sodium NLC and >100 nm sized phenytoin sodium NLC via the intranasal route. In the case of the plasma PK study, there is a 25–30-fold increase in the plasma AUC _0–∞_ of IV phenytoin sodium in comparison with <50 nm sized phenytoin sodium NLC. Since the quick cessation of seizure is highly essential for treating an acute epileptic fatal condition, rapid and direct brain drug delivery within 5 min is highly demanded to prevent further complications, which could be accomplished through smaller sized <50 nm phenytoin sodium NLC via intranasal olfactory epithelial route. The corresponding pharmacokinetic parameters are enlisted in Table 2.

**Figure 8:**
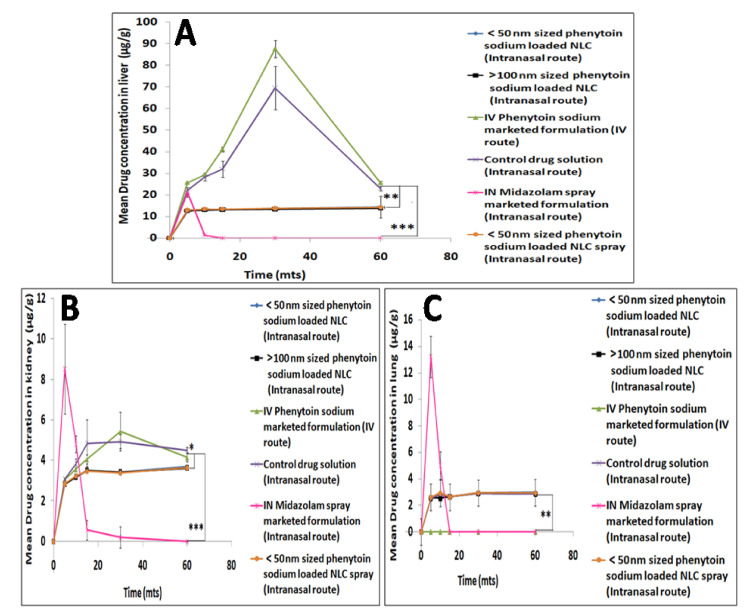
Mean liver (**A**), kidney (**B**) and lung (**C**) drug concentration profiles following intranasal administration of phenytoin sodium NLCs, control drug solution, midazolam spray marketed formulation and IV administration of phenytoin sodium marketed formulation. The level of statistical significance is expressed as a *p*-value; * indicates *p* < 0.05, ** indicates *p* < 0.01, *** indicates *p* < 0.001.

**Figure 9:**
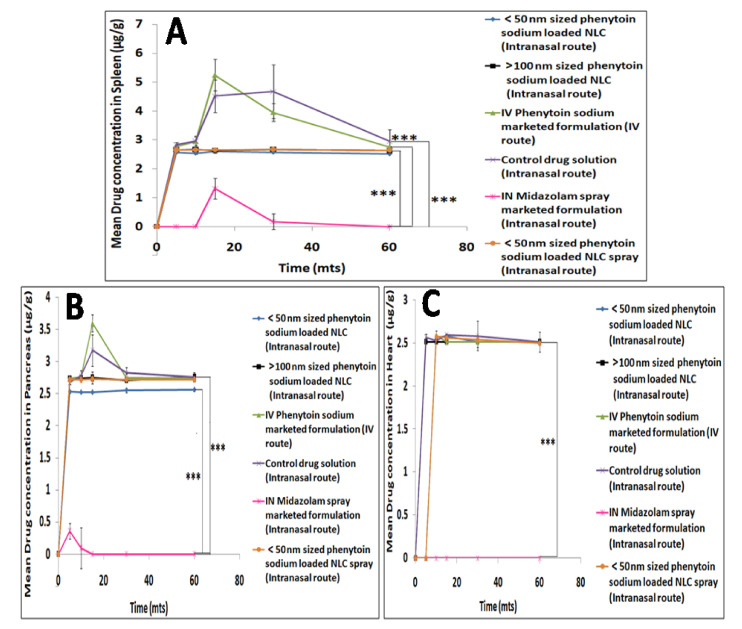
Mean spleen (**A**), pancreas (**B**) and heart (**C**) drug concentration profiles following intranasal administration of phenytoin sodium NLCs, control drug solution, midazolam marketed formulation and IV administration of phenytoin sodium marketed formulation. The level of statistical significance is expressed as a *p*-value; *** indicates *p* < 0.001.

Figure 8A–C showed the mean liver, kidney, lung concentrations and Figure 9A–C showed spleen, pancreas and heart concentrations of phenytoin sodium following intranasal administration of phenytoin sodium NLCs, control drug solution, midazolam spray marketed formulation and IV administration of phenytoin sodium marketed formulation performed in Wistar rats. Phenytoin was rapidly distributed into the liver following IV administration of phenytoin sodium and intranasal control drug solution with the highest concentrations of 87.60 μg/g and 69.51 μg/g, respectively, at the end of 30 min, which then decreased with time. However, the initial concentration of phenytoin in the liver was low at 5 min after administration of various sized intranasal NLC formulations and then increased with time reaching almost 14 μg/g at the end of 60 min. The higher accumulation of phenytoin sodium in the liver results in phenytoin hepatotoxicity, which is a serious idiosyncratic reaction associated with IV phenytoin therapy [46]. In fact, only a small amount of drug reached the liver from the smaller sized NLC via the intranasal olfactory route, which is comparable with that of intranasal midazolam spray marketed formulation. Thus, the study indicated that phenytoin sodium NLCs administered via the olfactory route does not produce any liver toxicity, which is highly beneficial in a clinical setup.

A maximum phenytoin concentration of 15.42 μg/g was obtained in the kidney by IV phenytoin sodium administration followed by intranasal control drug solution administration (4.93 μg/g) and various intranasal NLCs (3.3 μg/g), respectively, at the end of 30 min. The lungs received 0, 2.9 and 2.8 μg/g concentrations of phenytoin with the IV, intranasal control drug solution and various sized intranasal NLCs at the end of 30 min, respectively. The concentration of phenytoin in the lungs was comparable between the time points, with no significant difference for IV as well as various intranasal administrations. Over 60 min, phenytoin concentration in the heart remained steady (2.5 μg/g) with no significant difference between IV administration and intranasal phenytoin sodium NLCs. On the other hand, intranasal midazolam spray produced much lower drug concentrations in the heart compared to the IV solution. The spleen and pancreas tissues received comparatively higher concentrations of phenytoin following IV administration (5.24 and 3.59 μg/g, respectively) as well as intranasal administration of the control drug solution (4.5 and 3.17 μg/g, respectively). Over a 60min time interval, the concentrations of phenytoin in spleen and pancreas were comparable with no significant differences for the different sized NLC administrations. However, the intranasal phenytoin sodium NLCs and midazolam spray marketed formulation produced significantly lower concentration of drug in the spleen and pancreas than the corresponding intranasal control drug solution and IV phenytoin sodium marketed formulation.

The findings of the drug retention study in major peripheral organs confirmed that administering phenytoin sodium as smaller sized (<50 nm) intranasal nanolipid carriers considerably reduces phenytoin distribution in peripheral tissues compared to control drug solution as well as IV phenytoin sodium marketed formulation over for a period of 60 min. The study also revealed that systemic exposure of phenytoin can be reduced by administering it as smaller sized (<50 nm) intranasal nanolipid carriers. These results were consistent with the plasma-time concentrations of phenytoin.

**Figure 10:**
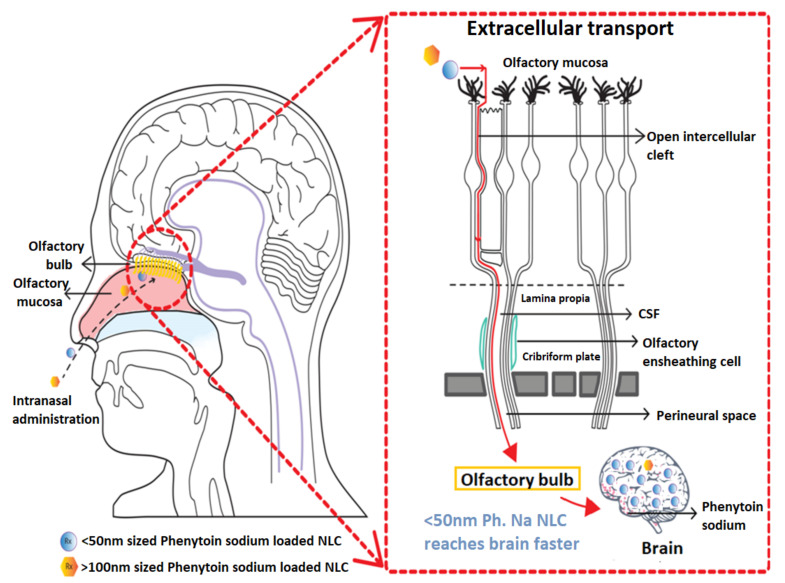
The schematic diagram showing direct nose to brain delivery of phenytoin sodium NLCs through olfactory epithelium by the extracellular perineural transport mechanism.

This is due to the fact that the intranasal <50 nm phenytoin sodium loaded NLC with sufficient lipophilicity would easily squeeze through the smaller gap between the olfactory cells to reach the lamina propia region of olfactory mucosa compared to larger sized >100 nm phenytoin sodium NLC. This extracellular or extra neuronal mechanism favors direct drug transport to the CSF or the brain parenchymal tissue within minutes following intranasal administration. This mechanism is also known as the olfactory epithelial pathway where the therapeutic agents are absorbed from the olfactory epithelium and diffused through the perineural channels to the CSF surrounding the brain via a perineural-convection mediated bulk flow transport mechanism [47]. The schematic diagram (Figure 10) shows the mechanism of the direct nose to brain drug delivery through the olfactory epithelial pathway. The results also revealed that the <50 nm sized phenytoin sodium NLC administered via the intranasal olfactory route is a safe and viable alternative for IV phenytoin sodium delivery. Consequently, through this intranasal olfactory epithelial route, the doses can be reduced by 5–10 times lesser than the clinically used parenteral dose for acute epileptic seizures [48,49].

### 3.8. In Vivo Nasal Toxicity Study

**Figure 11:**
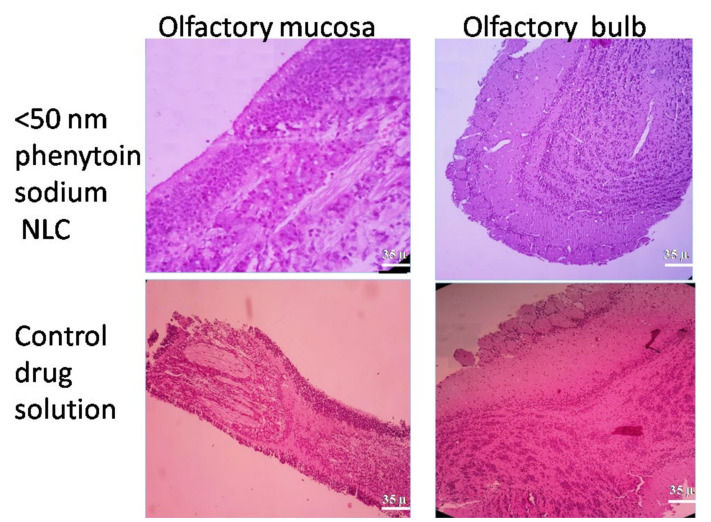
Histopathology images of rat’s nasal olfactory mucosa and olfactory bulb after treatment with <50 nm sized phenytoin sodium NLC as well as control drug solution.

The local toxic effect of the optimized NLC formulation, i.e., <50 nm sized phenytoin sodium NLC, was evaluated on both olfactory mucosa and olfactory bulb of Wistar rats and was compared with that of the control drug solution (drug in PBS pH 6.8) treated groups. The results are illustrated in Figure 11. Sections of <50 nm sized phenytoin sodium NLC group showing fragments lined by olfactory epithelium overlying submucosa with Bowman glands and lymphocytes and plasma cells are observed. Sections from the olfactory bulb showed normal morphology with outer nerve fibre layers, followed by glomerular layer, external plexiform layer, internal granular layer and central medulla, which was quite similar with that of the PBS treated groups. The results of the nasal histopathological studies indicated that there is no toxic impact on the microscopic structure of olfactory mucosa and olfactory bulb, as the surface epithelium lining and the granular cellular structure of the nasal mucosa and olfactory bulb were found completely intact [50].

## 4. Conclusions

In this work, we prepared and characterized the biocompatible Phenytoin sodium loaded NLCs of three different sizes (<50 nm, 50–100 nm and >100 nm) by melt emulsification method. The in vitro drug release profile indicated a complete drug release within 15 min for <50 nm sized NLCs compared to other sized NLCs. This immediate drug release is highly essential for the acute treatment of epilepsy. Further ex vivo permeation studies demonstrated higher permeation of drugs through nasal olfactory epithelium from <50 nm sized phenytoin sodium NLC with enhanced flux value compared to other NLCs as well as the control drug solution. This higher drug permeation of NLC through olfactory epithelium due to its small size as well as the lipidic nature is beneficial, whereby it reaches the brain rapidly and releases the drug within 15 min in order to produce a rapid onset of action. Furthermore, in vivo pharmacokinetic studies performed using Wistar rats showed higher drug accumulation in the CSF and brain tissues compared to other organs within 5 min for the <50 nm phenytoin sodium NLC administered via an intranasal olfactory route, which was deemed to be a safer route. However, IV phenytoin sodium marketed formulation showed greater accumulation of phenytoin sodium in the liver, indicating hepatic toxicity of drugs via IV route. Hence, it was concluded that <50 nm sized phenytoin sodium loaded NLCs could be an efficient carrier for the delivery of phenytoin sodium to treat acute epileptic seizures via the intranasal route while reducing peripheral toxicity. Although enhanced olfactory uptake of <50 nm size nano lipid carriers has been demonstrated in this study, further in vivo animal studies are essential to prove the antiepileptic potential of the developed phenytoin sodium NLCs.

## Figures and Tables

**Table 1:** Study design for in vivo pharmacokinetic study.

Group	Total No. of Animals	Animal Sub Groups Based on Different Time Intervals of Euthanasia					Treatment	Drug Amount	Dose Volume	Route
		5 min	10 min	15 min	30 min	1 h				
I	20	4	4	4	4	4	Vehicle (saline)	-	(50 µL/nostril) * 2	Intranasal
II	30	6	6	6	6	6	<50 nm sized phenytoin sodium loaded NLC	800 µg	(50 µL/nostril) * 2	Intranasal
III	30	6	6	6	6	6	>100 nm sized phenytoin sodium loaded NLC	800 µg	(50 µL/nostril) * 2	Intranasal
IV	30	6	6	6	6	6	Control drug solution (Drug in nasal pH buffer)	800 µg	(50 µL/nostril) * 2	Intranasal
V	30	6	6	6	6	6	Intranasal midazolam spray (marketed formulation)	800 µg	(80 µL/nostril) * 2	Intranasal
VI	30	6	6	6	6	6	Intravenous Phenytoin sodium (marketed formulation)	800 µg	16 µL	Tail vein
VII	30	6	6	6	6	6	<50 nm sized phenytoin sodium loaded NLC spray	800 µg	(50 µL/nostril) * 2	Intranasal

(* denotes multiplied by).

**Table 2:** Pharmacokinetic parameters estimated in plasma, CSF and brain using Win Nonlin^®^ software.

Formulation	Route	Organ/Compartment	C_max_ (µg/mL or µg/g)	T_max_ (mts)	AUC_0-∞_ (µg/mL·h or µg/g·h)	AUC_0-t_ (µg/mL·h or µg/g·h)	Extrapolated AUC (µg/mL·h or µg/g·h)	Elimination Rate Constant (k_e_) (h^−1^)	Half-Life (t_1/2_) (h)	Mean Residence Time (MRT) (h)
<50 nm sized phenytoin sodium loaded NLC	intranasal	Plasma	5.99 ± 3.2	15	5.05 ± 1.2	5.04 ± 1.3	2.17 ± 1.5	2.04 ± 1.3	0.33 ± 0.2	0.32 ± 0.10
		CSF	728.85 ± 4.7	10	211.17 ± 4.8	211.13 ± 3.6	0.04 ± 3.2	1014.06 ± 4.2	0.0006 ± 0.2	0.31 ± 0.13
		Brain	698.79 ± 6.3	10	487.28 ± 5.0	466.44 ± 4.8	0.77 ± 4.2	414.19 ± 4.6	0.001 ± 0.2	0.47 ± 0.1
>100 nm sized phenytoin sodium loaded NLC	intranasal	Plasma	19.48 ± 4.2	5	7.75 ± 1.5	7.75 ± 1.4	0.10 ± 1.6	38.20 ± 1.2	0.018 ± 0.3	0.19 ± 0.1
		CSF	477.32 ± 3.7	15	188.48 ± 2.6	188.26 ± 2.8	0.05 ± 1.9	136.71 ± 2.5	0.005 ± 0.02	0.31 ± 0.04
		Brain	684.97 ± 5.1	15	347.33 ± 4.9	347.33 ± 4.2	0.85 ± 3.3	192.66 ± 2.2	0.003 ± 0.2	1.48 ± 0.4
Control drug solution (Drug in nasal pH buffer)	intranasal	Plasma	509.06 ± 3.3	30	256.54 ± 3.5	256.54 ± 3.2	3.15 ± 2.1	2.03 ± 1.0	0.34 ± 0.1	0.55 ± 0.2
		CSF	9.66 ± 7.2	15	7.78 ± 5.0	7.78 ± 4.2	3.15 ± 2.3	0.20 ± 0.1	0.34 ± 0.2	4.97 ± 1.3
		Brain	35.51 ± 5.5	15	15.42 ± 4.8	15.42 ± 4.2	2.43 ± 1.4	4.27 ± 1.3	0.16 ± 0.2	0.44 ± 0.02
Intranasal midazolam spray (marketed formulation)	intranasal	Plasma	387.2 ± 4.1	5	50.64 ± 2.9	51.44 ± 2.2	0.006 ± 0.1	972.78 ± 3.1	0.0007 ± 0.01	0.11 ± 0.01
		CSF	375.08 ± 3.7	5	31.81 ± 5.4	32.11 ± 4.4	0.002 ± 0.02	942.3 ± 3.2	0.0007 ± 0.02	0.08 ± 0.02
		Brain	510.61 ± 5.7	5	43.30 ± 2.2	43.31 ± 1.9	0.0001 ± 0.02	1282.8 ± 3.3	0.0005 ± 0.01	0.08 ± 0.02
Intravenous Phenytoin sodium injection (marketed formulation)	intravenous	Plasma	333.52 ± 2.9	30	150.89 ± 5.2	155.06 ± 4.8	1.08 ± 0.2	22.82 ± 3.1	0.03 ± 0.02	0.46 ± 0.2
		CSF	12.92 ± 4.6	15	31.81 ± 4.5	8.03 ± 3.2	1.91 ± 0.4	3.06 ± 0.6	0.22 ± 0.2	0.42 ± 0.1
		Brain	10.80 ± 4.9	15	9.15 ± 2.1	9.57 ± 1.6	2.57 ± 0.4	3.59 ± 0.4	0.19 ± 0.2	0.86 ± 0.3
<50 nm sized phenytoin sodium loaded NLC spray	intranasal	Plasma	5.76 ± 2.9	15	5.0 ± 3.6	4.81 ± 3.2	1.81 ± 0.4	2.22 ± 0.7	0.30 ± 0.2	0.49 ± 0.1
		CSF	740.89 ± 4.3	10	220.72 ± 5.2	217.06 ± 5.0	0.04 ± 0.02	1072.2 ± 3.2	0.0006 ± 0.02	0.30 ± 0.12
		Brain	712.53 ± 7.6	10	492.45 ± 7.8	499.78 ± 7.2	0.74 ± 0.3	451.26 ± 4.3	0.001 ± 0.02	0.45 ± 0.04

## Data Availability

Not applicable.

## References

[1] Trinka E., Cock H., Hesdorffer (2015). A definition and classification of status epilepticus—Report of the ILAE task force on classification of status epilepticus. Epilepsia.

[2] Holsti M., Dudley N., Schunk J., Adelgais K., Greenberg R., Olsen C., Healy A., Firth S., Filloux F. (2010). Intranasal midazolam vs. rectal diazepam for the home treatment of acute seizures in pediatric patients with epilepsy. Arch. Pediatr. Adolesc. Med..

[3] Glauser T., Shinnar S., Gloss D., Alldredge B., Arya R., Bainbridge J., Bare M., Bleck T., Dodson W.E., Garrity L. (2016). Evidence-based guideline: Treatment of convulsive status epilepticus in children and adults: Report of the Guideline Committee of the American Epilepsy Society. Epilepsy Curr..

[4] Alvarez V., Januel J.M., Burnand B., Rossetti A.O. (2011). Second-line status epilepticus treatment: Comparison of phenytoin, valproate, and levetiracetam. Epilepsia.

[5] Desta Z., Zhao X., Shin J.G., Flockhart D.A. (2002). Clinical significance of the cytochrome P450 2C19 genetic polymorphism. Clin. Pharmacokinet..

[6] Mc Kindley D.S., Boucher B.A., Hess M.M., Rodman J.H., Feler C., Fabian T.C. (1997). Effect of acute phase response on phenytoin metabolism in neurotrauma patients. J. Clin. Pharmacol..

[7] Nation R.L., Evans A.M., Milne R.W. (1990). Pharmacokinetic drug interactions with phenytoin (Part I). Clin. Pharmacokinet..

[8] Gallop K. (2010). Review article: Phenytoin use and efficacy in the ED. Emerg. Med. Australas.

[9] Appleton R.E., Gill A. (2003). Adverse events associated with intravenous phenytoin in children: A prospective study. Seizure.

[10] Kandimalla K.K., Donovan M.D. (2005). Transport of hydroxyzine and triprolidine across bovine olfactory mucosa: Role of passive diffusion in the direct nose-to-brain uptake of small molecules. Int. J. Pharm..

[11] Warnken Z.N., Smyth H.D.C., Watts A.B., Weitman S., Kuhn J.G., Williams R.O. (2016). Formulation and device design to increase nose to brain drug delivery. J. Drug Deliv. Sci. Technol..

[12] Wang D., Gao Y., Yun L. (2006). Study on brain targeting of raltitrexed following intranasal administration in rats. Cancer Chemother. Pharmacol..

[13] Jadhav K.R., Gambhire M.N., Shaikh I.M., Kadam V.J., Pisal S.S. (2007). Nasal drug delivery system-factors affecting and applications. Curr. Drug Ther..

[14] Ghasemiyeh P., Mohammadi S.S. (2018). Solid lipid nanoparticles and nanostructured lipid carriers as novel drug delivery systems: Applications, advantages and disadvantages. Res. Pharm. Sci..

[15] Pardeshi C.V., Belgamwar V.S. (2013). Direct nose to brain drug delivery via integrated nerve pathways bypassing the blood–brain barrier: An excellent platform for brain targeting. Expert Opin. Drug Deliv..

[16] Md S., Khan R.A., Mustafa G., Chuttani K., Baboota S., Sahni J.K., Ali J. (2013). Bromocriptine loaded chitosan nanoparticles intended for direct nose to brain delivery: Pharmacodynamic, pharmacokinetic and scintigraphy study in mice model. Eur. J. Pharm. Sci..

[17] Bourganis V., Kammona O., Alexopoulos A., Kiparissides C. (2018). Recent advances in carrier mediated nose-to-brain delivery of pharmaceutics. Eur. J. Pharm. Biopharm..

[18] Espinoza L.C., Silva-Abreu M., Clares B., Rodríguez-Lagunas M.J., Halbaut L., Cañas M.A., Calpena A.C. (2019). Formulation strategies to improve nose-to-brain delivery of donepezil. Pharmaceutics.

[19] Wong H.L., Wu X.Y., Bendayan R. (2012). Nanotechnological advances for the delivery of CNS therapeutics. Adv. Drug Deliv. Rev..

[20] Gänger S., Schindowski K. (2018). Tailoring formulations for intranasal nose-to-brain delivery: A review on architecture, physico-chemical characteristics and mucociliary clearance of the nasal olfactory mucosa. Pharmaceutics.

[21] Varshosaz J., Eskandari S., Tabakhian M. (2010). Production and optimization of valproic acid nanostructured lipid carriers by the Taguchi design. Pharm. Dev. Technol..

[22] Shefrin S., Sreelaxmi C.S., Vishnu V., Sreeja C.N. (2019). Antiepileptic drug loaded niosomal transdermal patch for enhanced skin permeation. Int. J. Appl. Pharm..

[23] Varshosaz J., Tabbakhian M., Mohammadi M.Y. (2010). Formulation and optimization of solid lipid nanoparticles of buspirone HCl for enhancement of its oral bioavailability. J. Liposome Res..

[24] Bonaccorso A., Musumeci T., Serapide M.F., Pellitteri R., Uchegbu I.F., Puglisi G. (2017). Nose to brain delivery in rats: Effect of surface charge of rhodamine B labeled nanocarriers on brain subregion localization. Colloids Surf. B Biointerfaces.

[25] Li Q., Cai T., Huang Y., Xia X., Cole S.P.C., Cai Y. (2017). A Review of the Structure, Preparation, and Application of NLCs, PNPs and PLNs. Nanomaterials.

[26] Kovacevic A., Savic S., Vuleta G., Müller R.H., Keck C.M. (2011). Polyhydroxy surfactants for the formulation of lipid nanoparticles (SLN and NLC): Effect on size, physical stability and particle matrix structure. Int. J. Pharm..

[27] Moein M., Amir A., Reza H., Soliman M.S. (2020). Nose-to-brain delivery of sumatriptan- loaded nanostructured lipid carriers: Preparation, optimization, characterization and pharmacokinetic evaluation. J. Pharm. Pharmacol..

[28] Jain K., Sood S., Gowthamarajan K. (2015). Optimization of artemether-loaded NLC for intranasal delivery using central composite design. Drug Deliv..

[29] Dunston D., Ashby S., Krosnowski K., Ogura T., Lin W. (2013). An effective manual deboning method to prepare intact mouse nasal tissue with preserved anatomical organization. J. Vis. Exp..

[30] Östh K., Gråsjö J., Björk E. (2002). A new method for drug transport studies on pig nasal mucosa using a horizontal Ussing chamber. J. Pharm. Sci..

[31] Asha P., Fathima K.M., Sreeja C.N. (2017). Intra nasal *in situ* gelling system of lamotrigine using ion activated mucoadhesive polymer. Open J. Med. Chem..

[32] Sabitha M., Rejinold N.S., Nair A., Lakshmanan V.K., Nair S.V., Jayakumar R. (2013). Development and evaluation of 5-fluorouracil loaded chitin nanogels fortreatment of skin cancer. Carbohydr. Polym..

[33] Mangalathillam S., Rejinold N.S., Nair A., Lakshmanan V.K., Nair S.V., Jayakumar R. (2012). Curcumin loaded chitin nanogels for skin cancer treatment via thetransdermal route. Nanoscale.

[34] Liu J.S., Wang J.H., Zhou J., Tang X.H., Xu L., Shen T., Wu X.Y., Hong Z. (2014). Enhanced brain delivery of lamotrigine with PluronicP123-based nanocarrier. Int. J. Nanomed..

[35] Rajitha P., Jayakumar R., Sabitha M. (2017). Comparative anti-psoriatic efficacy studies of clobetasol loaded chitin nanogel and marketed cream. Eur. J. Pharm. Sci..

[36] Zhang Q.Z., Jiang X.G., Wu C.H. (2004). Distribution of nimodipine in brain following intranasal administration in rats. Acta Pharmacol. Sin..

[37] Rohini G.M., Hitendra S.M. (2016). Curcumin-loaded nanostructured lipid carriers (NLCs) for nasal administration: Design, characterization and in vivo study. Drug Deliv..

[38] Jain R., Nabar S., Dandekar P., Patravale V. (2010). Micellar nanocarriers: Potential nose-to-brain delivery of zolmitriptan as novel migraine therapy. Pharm. Res..

[39] Severino P., Pinho S.C., Souto E.B., Santana M.H. (2011). Polymorphism, crystallinity and hydrophilic-lipophilic balance of stearic acid and stearic acid–capric/caprylic triglyceride matrices for production of stable nanoparticles. Colloids Surf. B Biointerfaces.

[40] Sonvico F., Clementino A., Buttini F., Colombo G., Pescina S., Stanisçuaski Guterres S., Raffin Pohlmann A., Nicoli S. (2018). Surface-modified nanocarriers for nose-to-brain delivery: From bioadhesion to targeting. Pharmaceutics.

[41] Kumar M., Misra A., Babbar A.K., Mishra A.K., Mishra P., Pathak K. (2008). Intranasal nanoemulsion based brain targeting drug delivery system of risperidone. Int. J. Pharm..

[42] Wu C., Li B., Zhang Y., Chen T., Chen C., Jiang W., Wang Q., Chen T. (2020). Intranasal delivery of paeoniflorin nanocrystals for brain targeting. Asian J. Pharm. Sci..

[43] Mistry A., Stolnik S., Illum L. (2015). Nose-to-brain delivery: Investigation of the transport of nanoparticles with different surface characteristics and sizes in excised porcine olfactory epithelium. Mol. Pharm..

[44] Davda J., Labhasetwar J.V. (2002). Characterization of nanoparticle uptake by endothelial cells. Int. J. Pharm..

[45] Selvaraj K., Gowthamarajan K., Karri V.V. (2018). Nose to brain transport pathways an overview: Potential of nanostructured lipid carriers in nose to brain targeting. Artif. Cells Nanomed. Biotechnol..

[46] Maureen A.S., Greg S.U. (1989). Phenytoin hepatotoxicity: A review of the literature. Ann. Pharm..

[47] Jeffrey J.L., Robert G. (2012). Thorne. Intranasal delivery of biologics to the central nervous system. Adv. Drug Deliv. Rev..

[48] Rajput A.P., Butani S.B. (2019). Resveratrol anchored nanostructured lipid carrier loaded in situ gel via nasal route: Formulation, optimization and in vivo characterization. J. Drug Deliv. Sci. Technol..

[49] Gadgil P., Shah J., Chow D.L. (2018). Enhanced brain delivery with lower hepatic exposure of lazaroid loaded nanostructured lipid carriers developed using a design of experiment approach. Int. J. Pharm..

[50] Zhao Y.Z., Li X., Lu C.T., Lin M., Chen L.J., Xiang Q., Zhang M., Jin R.-R., Jiang X., Shen X.T. (2014). Gelatin nanostructured lipid carriers-mediated intranasal delivery of basic fibroblast growth 1328 factor enhances functional recovery in hemiparkinsonian rats. Nanomedicine.

